# Gas Chromatography-Ion Mobility Spectrometry Detection of Odor Fingerprint as Markers of Rapeseed Oil Refined Grade

**DOI:** 10.1155/2019/3163204

**Published:** 2019-08-04

**Authors:** Tong Chen, Xingpu Qi, Mingjie Chen, Bin Chen

**Affiliations:** ^1^School of Food and Biological Engineering, Jiangsu University, Zhenjiang 212013, China; ^2^Jiangsu Agri-animal Husbandry Vocational College, No. 8 East Phoenix Road, Taizhou, Jiangsu 225300, China

## Abstract

In this work, gas chromatography-ion mobility spectrometry (GC-IMS) was used to analyze the volatile organic compound changes of rapeseed oil with different refined grades, the odor fingerprints of refined rapeseed oil were constructed, and a nonlinear model was built to realize rapid and accurate discrimination of rapeseed oil with different refined grades. 124 rapeseed oil samples with different refined grades were collected and analyzed by GC-IMS and chemometric tools, and 34 characteristic peaks were selected by the colorized difference method as variables to characterize the internal quality in rapeseed oil of different refined grades. The principal component analysis algorithm was used to further reduce dimensionality and extract the most relevant information. The *k*-nearest neighbor algorithm was applied to build a discriminant model. All the samples were recognized accurately without errors, and the results show the potential of this method to discriminate different refined grades of vegetable oil.

## 1. Introduction

With pleasant flavor and taste, rapeseed oil has become a popular edible vegetable oil and is often used as a flavor enhancer in China and many other Asian countries [1]. Moreover, rapeseed oil contains various nutritional compounds such as lignans, tocopherols, and unsaturated fatty acids, which are beneficial to human health [2–4]. The volatile organic components (VOCs) in edible vegetable oil are mainly alcohols, aldehydes, and ketones. Different varieties of vegetable oil contain different kinds and contents of odor components, and even oil of the same variety retain different volatile components due to various processes of oil refining. A critical phase of the edible oil production chain is the final refining aimed at removing free fatty acids (in too high concentrations), which leads to the rancidity of the oil and other minor components such as phospholipids, pigments, proteins, oxidation products, and possible residue of the solvent used for extraction. Edible vegetable oil produced by different processes (e.g., cold pressing and solvent extraction) have different prices, especially for vegetable oil with different refining process, which could produce a great commercial problem because high-level refining oil has several times higher price than low-level one [5]. Therefore, the scientific distinction of vegetable oil with different refinement degrees is a new problem faced by quality inspection technicians and quality control personnel of production enterprises [6].

Traditional analytical methods have been used to determine the refinement grades of oil on the basis of its physical or chemical properties [7]. The traditional methods commonly used in industry for assessing vegetable oil quality grading is the human olfactory sense, especially for the smell and taste index. However, the cost of employing trained sensory experts is relatively high since they can only work for a short period of time due to sensory fatigue. On the contrary, sensory evaluation has limitation on reproducibility and repeatability of results, which is not normally used for quantitative analysis. The physicochemical properties of vegetable oil can also be determined by using instrumental techniques, e.g., gas chromatography (GC) [8], gas chromatography-mass spectrometry (GC-MS) [9], and high-performance liquid chromatography (HPLC) [10]. The requirement for tedious extraction, long analysis time, and special environment significantly limited wide use of these chromatographic methods. Furthermore, many indexes of oil quality need detection, like refractive index, iodine value, saponification value, and so on. For spectroscopy methods, Fourier transform infrared (FTIR), near-infrared reflectance spectroscopy (NIRS), and Raman spectrum have been shown to be useful for vegetable oil quality analysis [11]. However, the analysis of the obtained data requires complex algorithms and special software, which make it difficult for ordinary inspectors to master. Electronic nose is also regarded as a green technique in this field [12]. Unfortunately, it should be noted however that, at the current stage of development, electronic noses have certain limitations on sensor drift, longevity, and sensitivity in certain cases [13]. For these reasons, there is still a need to develop a more effective technique which could supply the human olfactory sense and to be used to obtain sensory data within a short period of time and at a low cost.

Ion mobility spectrometry (IMS) is an analytical technique for the detection of trace gases [14–17]. A gas-phase sample is ionized by chemical ionization in the drift gas (such as nitrogen, clean air, and helium) in the positive or negative mode. The analyte ions are accelerated towards the detector (a Faraday plate) in a constant electric field. During their drift towards the detector, the ions move with a constant velocity due to an equilibrium between acceleration by the electric field and deceleration by collisions with the drift gas molecules. The drift time of ions from the shutter grid to the detector depends on their mass and structure and is hence characteristics of analytes. However, for complex samples, the separation performance is limited and the application of IMS with direct sample introduction will not be sufficient, as gathering analytes might take place in the ionization chamber and drift region. Thus, some authors [18–21] have used chromatographic columns prior to ion mobility analysis and have proved that the coupling of a GC column as previous separation step to IMS can significantly improve the results. In recent years, GC-IMS has proved suitable for metabolic profiling of human breath [22], process and quality control analysis [23–26], as well as food quality and safety control [26–30]. IMS instrument combined with a GC column allows original mobility spectra to be automatically acquired upon chromatographic elution of each target compound, and several spectra at a given retention time can be processed in order to obtain twice the information (retention time and drift time), which provide larger amounts of analytical information from each sample.

In this study, GC-IMS-based volatile organic compounds were utilized to evaluate the vegetable oil refining process. Four different grades of rapeseed oil samples were collected. The colorized difference method was applied to discover characteristic VOC markers of different refined grades of oil. And principal component analysis (PCA) and k-nearest neighbor (kNN) were used to build the qualitative discrimination model. Compared to classical techniques, this approach has the potential to be a faster, more accurate, and cheaper recognition tool for discriminating different refined grades of each type of edible oil samples and might provide a reference for grade identification of vegetable oil refinery.

## 2. Materials and Methods

### 2.1. Oil Samples

Four different refined grades of commercially available rapeseed oil samples were collected from the Wilmar Global Research and Development Center (Shanghai). The raw materials were obtained from local producing areas in Jiangsu Province. All the oil samples were extracted from rapeseed seeds by traditional solvent extraction, and the refining processes mainly contained degumming, deacidification, bleaching, dewaxing, and deodorization technology in order to realize long-term storage or act as edible oil products. The refined grades were discriminated by the Chinese National Standard GB/T 1536-2004. The sample acquisition cycle lasted about six months, and finally, a total of 124 samples were obtained, among which 31 samples were labeled grade 1, 34 samples were marked grade 2, 26 samples were signed grade 3, and 33 samples were of grade 4. Initially, the collected samples in batches were stored at −5°C in the refrigerator in order to get sufficient samples. All the samples were brought to room temperature for 10 min and were homogenized with vortex for 60 s before detection.

### 2.2. Experiment Device

All prepared oil samples were analyzed with the commercial GC-IMS device (FlavourSpec®) from G.A.S. (Gesellschaft für Analytische Sensorysteme GmbH, Dortmund, Germany). The device was equipped with an automatic sampler unit (CTC-PAL, CTC Analytics AG, Zwingen, Switzerland) for 32 vials and furnished with a 1 mL Hamilton syringe, a heated splitless injector with 2 mm ID, 6.5 mm OD × 78.5 mm fused quartz glass, and a radioactive ionization source (tritium) of 6.5 KeV.

Each rapeseed oil sample (2 mL) was placed in a 20 mL vial and closed with magnetic screw caps. After 10 min of incubation at 90°C, 200 *μ*L of headspace was automatically injected by the heated syringe (90°C) into the heated injector (95°C) of the GC-IMS instrument. The separation was performed using a nonpolar column constituted by 94% methyl-5% phenyl-1% vinyl silicone with a 30 m of length. After that, the VOCs were pushed into the GC column (40°C) through a carrier gas (N_2_, purity ≥99.999%). The carrier gas flow was initially set at 2 mL·min^−1^ during 2 min, and then the flow was linearly increased to 15 mL·min^−1^ within 8 min; next, it was raised to 100 mL·min^−1^ within 10 min, and finally, the flow reached 150 mL·min^−1^ in the next 10 min. The total run time was 30 min in order to get a better separation effect. After the separation in the capillary column at 40°C, the headspace was pushed into the ionization chamber for ionization prior, then driven into the drift region via a shutter grid, and finally passed into the IMS detector. The drift tube was 10 cm long. It was operated at a constant voltage of 400 V·cm^−1^ and a temperature of 45°C. The drift gas (N_2_, purity ≥99.999%) flow was set at 150 mL·min^−1^. The ion mode was made with the positive mode. Each spectrum was obtained by the average of 32 scans, the grid pulse width of 100 *μ*s, the sampling frequency of 150 kHz, and the repetition rate of 21 ms.

### 2.3. Colorized Difference

A popular method for comparing two matrices is to form a difference image by subtracting the individual intensity values of one matrix from the corresponding intensity values of the other matrix [31]. In this condition, a positive difference indicates that the analyzed matrix has a larger element value and a negative difference indicates that the reference matrix has a larger element value. The difference image can be displayed with a grayscale so that medium gray represents zero difference, brighter values represent positive differences, and darker values represent negative differences. In order to make the differences more apparent and to retain some context for those differences, the original grayscale difference method is modified to color code the differences and incorporate the comparison image pixel intensities [32].

### 2.4. Data Analysis

Data of samples were obtained by IMS Control TFTP Server Software. The identification of specific volatile compounds was realized by the software GC×IMS Library Search version 1.0.3. They were obtained from G.A.S (Dortmund, Germany). In addition, data display, feature extraction, and assessment of the ion mobility profiling were carried out using MATLAB R2009a software (The Mathworks Inc., Natick, USA) and PRTools 5.0 toolkit (Delft University of Technology, Netherlands).

The multidimensional signals of raw data required pretreatment before statistical analysis with a view to avoid possible variations among samples and a resulting misidentification. Firstly, the data were normalized with respect to the RIP (peak corresponding to the reactant ions or hydrated protons required to ionize the analytes) intensity (as an internal standard). Secondly, the region of each topographic plot was selected by limiting the retention time from 99.06 to 604.5 s and drift time from 0.885 to 1.6739 ms on the basis of containing the majority of the data of each topographic plot. After that, the colorized difference method was used to observe the differences of the VOCs from different refined grades. Then, since GC-IMS analysis resulted in a 3D graph in which each analyte is represented by a peak (spot) that is characterized by the retention time (*y* axis), drift time (*x* axis), and the intensity of the signal, a series of characteristic spot was selected as profiling markers based on the changes of VOCs. Once the pretreatment was performed on both samples, the selected characteristic spots were arranged as variables to obtain the dataset used in the chemometric treatment. Next, PCA was employed for dimensionality reduction and extraction of the most relevant information. Finally, a kNN classifier was applied for qualitative discrimination between different refined grades of oil.

## 3. Results and Discussion

### 3.1. Analysis of VOC Differences

**Figure 1:**
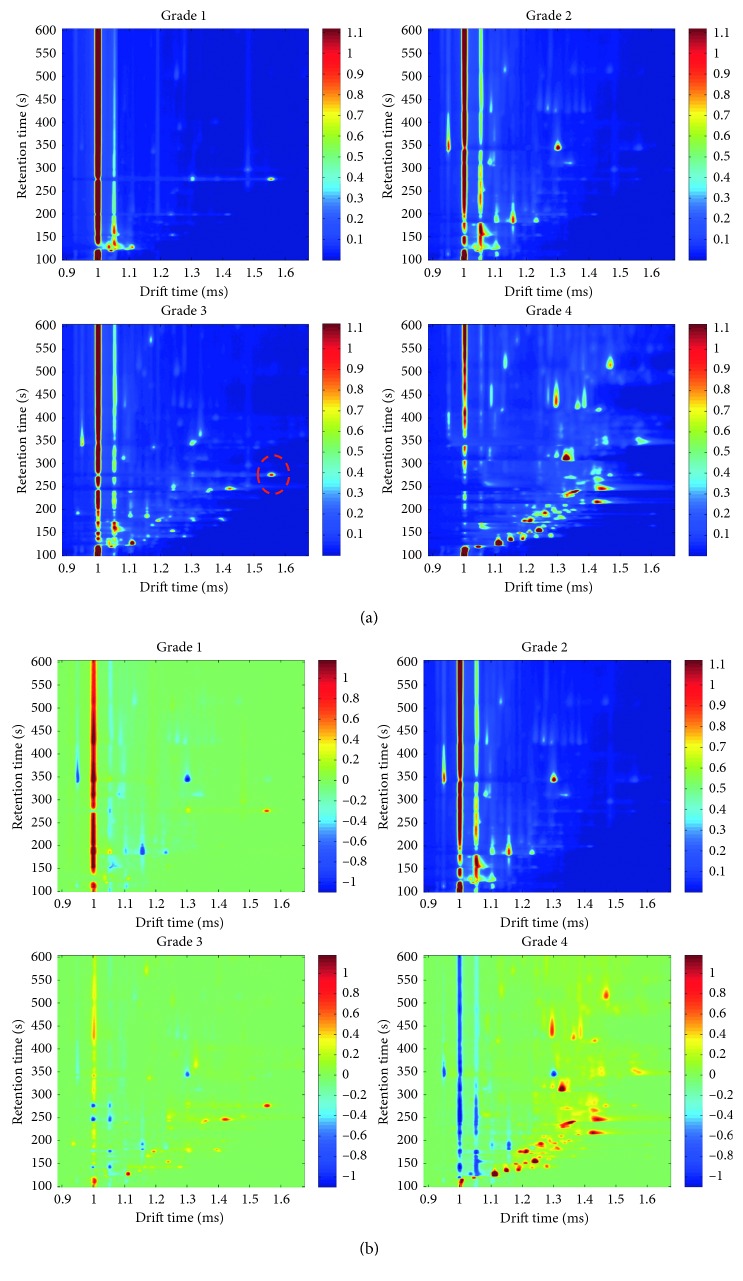
GC-IMS original spectrum (a) and colorized difference plot (b) of refined grades of rapeseed oil.

As mentioned above, since the original raw data of each sample was large (4615 × 4500), the obtained matrix of each sample was cut apart into the subset (1297 × 952) by limiting the retention time from 99.06 to 604.5 s and drift time from 0.885 to 1.6739 ms on the basis of retaining the major information. Then, one sample was randomly chosen from each refined level of rapeseed oil samples, and the three-dimensional plot of GC-IMS is shown in Figure 1(a). The title of each subgraph in Figure 1 formed with four refining levels (grade 1, grade 2, grade 3, and grade 4) of rapeseed oil, respectively. As shown in Figure 1(a), grade 4 rapeseed oil apparently had more VOCs and the corresponding concentrations were higher, where grade 1 rapeseed oil had few compounds and relatively weak peak intensity. With the increase of refined degree of rapeseed oil, it can be inferred that the number of VOCs in the vegetable oil shows decreasing trend and the concentration was also weakened. On the contrary, new VOCs are also produced in the oil samples of different refined grades, accompanied by the disappearance of the original substance (e.g., region marked by a red dotted ellipse).

### 3.2. Characteristic VOC Markers for Different Refined Grades

**Figure 2:**
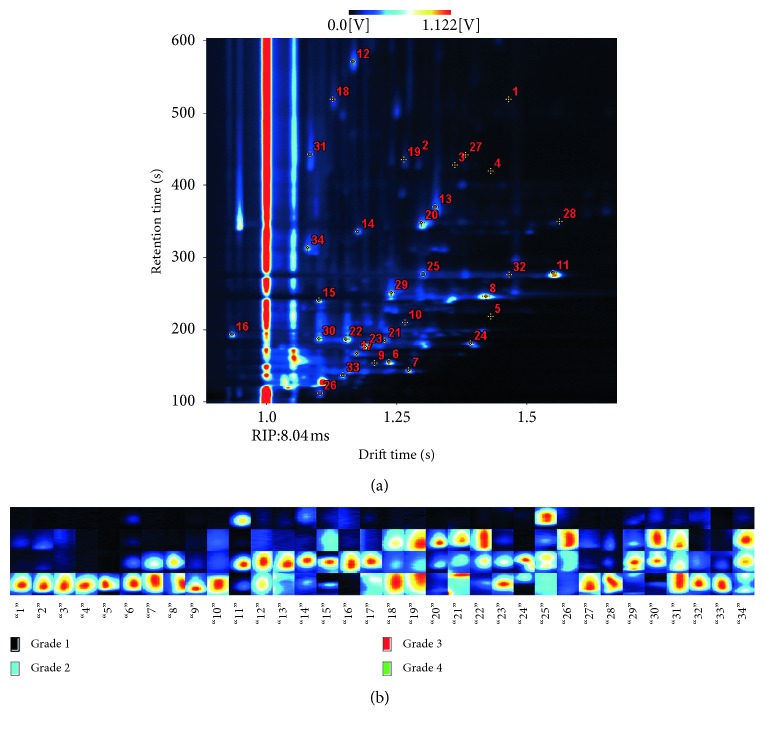
34 characteristic peaks' (spot) selection locations (a) and overview report (b) of the peaks.

In order to observe the changes of VOCs in rapeseed oil with different refined grades intuitively, the colorized difference method was applied to select a number of characteristic peaks. Firstly, each grade of rapeseed oil samples was calculated by a cumulative sum and further its average value was also computed based on the individual intensity values. Then, four average GC-IMS spectra (four matrices) were obtained, which characterized specific VOC information of each refined grade oil. Next, one of the matrices was selected as a reference, and difference matrices (the rest three matrices) were obtained by subtracting the individual value of the reference matrix and the colorized difference method accomplished by the MATLAB code used to display the visual results. Finally, an area set was created by integrating all the characteristic peaks based on color changes.  Figure 1(b) shows the difference matrix plot of average GC-IMS spectra. As shown in Figure 1(b), the grade 2 average matrix was selected as a reference and the others were displayed by a colorized difference. The red region indicated that the sample had more volatile compounds compared with the reference sample. The deeper the color, the more the concentration it had, and the blue region was the opposite. Based on this principle, 34 characteristic peaks were selected as variables to present the oil quality and positions of the selected peaks are shown in Figure 2(a). An area set able to integrate all the markers peaks was created for view intuitively (shown in Figure 2(b)), which was applied for the analysis of all the samples considered within this study and for model set up.

As shown in Figure 2(b), *x* axis was marked the characteristic spots and *y* axis was the sample type. For grade 1 rapeseed oil samples, there were only three characteristic components (markers 11, 14, and 25). Combined with the previous analysis, it can be inferred that the higher the level of vegetable oil refining, the fewer the VOCs it had. Furthermore, some new compounds were produced and disappeared later (e.g., markers 11, 14, 15, 16, 17, 29, and 30) in the process of improving oil refining. It was easy to find that different refined grades of rapeseed oil samples had their specific compounds, which implied the possibility of discrimination based on the volatile organic compounds. The chemical compound information of partial characteristic peaks was retrieved by using GC-IMS Library Search software, which is shown in Table 1.

### 3.3. Multivariate Analysis

It was very easy to view that there were obvious differences between refined grades of rapeseed oil samples. However, it was difficult to realize digital expression. Therefore, chemometric tools were needed to conduct further analysis, which had been proved to be useful and powerful for data analysis.

**Figure 3:**
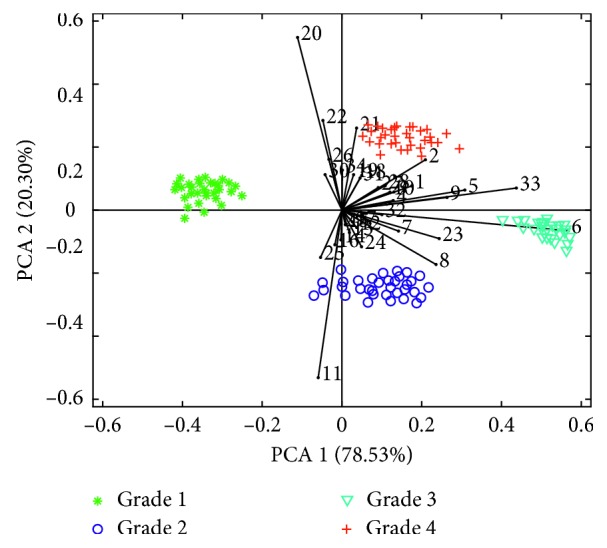
Principal component scores and load plot.

Thirty-four selected characteristic peaks were used as variables (height of peak) to form a matrix, and the PCA algorithm was applied to process and analyze the formed matrix. Principal component scores obtained were sorted from high to low according to the cumulative contribution rate, and the first 2 principal component score matrices were used to show the cluster of oil samples with different refined grades. As shown in Figure 3, the data were mapped on two most important principal components PC1 and PC2. The axis heading in each figure was labeled with the respective contribution rates of PC1 and PC2 after the PCA process. As can be seen, PC1 and PC2 explained 98.83% of the original information, which meant that the first 2 PCs could give the most information of the dataset. As shown, each refined grade of oil samples had its own cluster group and four grades of oil samples were well distinguished. At the same time, the loading matrix (the black line and corresponding peak markers in Figure 3) was also visualized. The loading matrix is the projection of features on principal components, which can be used to study the correlation and importance between different features. As shown in Figure 3, compared with other signs, the features of characteristic peaks (markers 2, 5, 6, 8, 9, 11, 20, 21, 22, 23, and 33) were more important compounds because their positions were far from the coordinate origin. As can be observed, only a few characteristic variables could be used to distinguish grade 2, grade 3, and grade 4 rapeseed refined oil rapidly (e.g., markers 8 and 33). However, there were relatively few feature variables between grade 1 and grade 2 oil samples, and the feature variables were close to the origin of the coordinate system and of less importance, which could not be used to distinguish grade 1 and grade 2 refined oil samples. Therefore, it was necessary to further use chemometric tools to establish a model for recognition.

**Figure 4:**
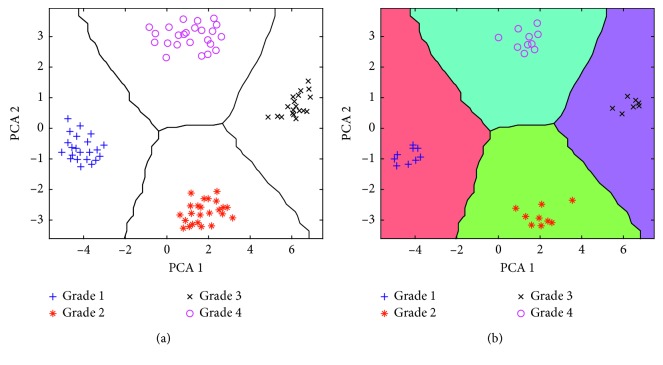
Classification results of the model from the training set (a) and testing set (b).

The kNN algorithm is a simple technique to generate nonlinear boundaries between classes. kNN finds the closest *k* samples of the training dataset to the unknown sample and assigns the predominant class to it [33]. The scoring matrices of the former two principal components were selected as input variables, and a kNN classifier was applied to find out the percentage of the correct classification of the chemometric model. Before building the model, 70% of the samples were randomly selected to construct the calibration model and the rest 30% samples were used to evaluate it. The accuracy of classification was used as an indicator of the performance of a classifier. The results obtained are shown in Figure 4. As can be observed, both the training set (Figure 4(a)) and testing set (Figure 4(b)) obtained a good classification, and all the samples were distinguished to the right categories without any errors. Therefore, the method developed in this study verifies that different refined grades of rapeseed oil can be determined by GC-IMS and chemometrics.

## 4. Conclusions

In this paper, GC-IMS has been proposed to analyze the VOCs differences of the rapeseed oil with different grades. 124 samples were detected, and the characteristic peaks were extracted and analyzed by the colorized difference method and PCA algorithm. A combination of these methods was proved to be useful for feature extraction. Moreover, a kNN recognition model was constructed, and the results showed a good classification of rapeseed oil with different refined grades.

The methodology developed has a capability to distinguish different refined grades of vegetable oil. The analysis time only needs about 30 min, which is greatly less than the traditional techniques, and no sample pretreatment is required. Therefore, GC-IMS can be seen as a powerful authentication method with chemometrics and can also be spread and applied in other areas.

## Figures and Tables

**Table 1:** Compounds' information corresponding to the characteristic peaks.

Marker number	Compound	Retention time (s)	Drift time (ms)
1	Butyl hexanoate	521.292	1.466
2	Diethyl butanedioate	445.647	1.2954
3	(E,Z)-2,6-nonadienal	429.143	1.3641
6	Pentanoic acid	156.137	1.2357
7	Ethyl pentanoate	144.446	1.2728
8	2-Ethyl-1-hexanol	246.91	1.4193
11	Alpha-phenylethanol alcohol	277.167	1.5522
12	2,6-Dichlorophenol	572.867	1.1647
14	Acetophenone	337.683	1.176
19	2,3-Diethyl-5-methylpyrazine	436.708	1.265
20	Dibutyl sulfide	349.373	1.2965
21	Alpha-pinene	187.082	1.2278
22	Benzaldehyde	187.77	1.1523
24	5-Methyl-2-furancarboxaldehyde	181.919	1.3919
25	Limonene	278.035	1.3017
28	Acetophenone	350.951	1.5608
33	Cyclohexanone	136.347	1.1476

## Data Availability

All the data used to support the findings of this study are available from the first author upon request.
